# Protecting endemic species from African Catfish invasion when community behavioral responses get in the way

**DOI:** 10.1371/journal.pone.0209009

**Published:** 2018-12-27

**Authors:** Ram Ranjan

**Affiliations:** Department of Environmental Sciences, Faculty of Science and Engineering, Macquarie University, Sydney, Australia; Hellenic Agricultural Organization - Demeter, GREECE

## Abstract

Exotic invasive fish species, when introduced into pristine natural environments, threaten the survival of many endemic species. Management challenges associated with controlling their further spread and protecting endemic species can be exacerbated when the same exotic fish species also provide gastronomical benefits to humans. Local human populations can switch their consumption preferences toward the exotic fish species, leading to an increase in their spread rate and control costs. Using the example of the African Catfish invasion in a freshwater lake, we develop a bioeconomic model of its optimal control, which also incorporates the behavioral challenges arising from a gastronomical preference for the exotic fish species. In particular, the cost of catfish control increases with its consumption demand, which, through altering the inter-species dynamics, threatens the survival of endemic fish species. The manager has at his disposal the market and non-market values of the endemic fish species to invest toward their preservation efforts. The non-market value of the endemic species is further modeled as endogenous to the community’s preference switching. Results suggest that a late detection of the exotic fish species in freshwater bodies can increase their control costs enough to make their eradication challenging, especially when the manager faces financial resource constraints. The presence of behavioral effects adds to this challenge — directly, through increasing the control costs, and indirectly, through lowering the non-market value of the endemic fish species.

## Introduction

Exotic species invasion, in combination with habitat degradation, poses significant threats to global biodiversity through causing large-scale extinction of endemic species [1]. The challenge of invasive species management can become complicated in situations where such species also provide some economic benefits, such as supplementing the livelihoods of farmers or fishermen. For example, the African Catfish (*Clarias Geriepinus*), which threatens the survival of multiple endemic species globally, has been difficult to eradicate due to its increasing preference and commercial breeding for consumption purposes [2].

Commercial breeding of the catfish increases the risk of its spread into natural water bodies. However, the economic rewards from commercial breeding of catfish are far higher compared to endemic species, given the former’s ability to grow rapidly (yielding more weight per fish) irrespective of the quality of the habitat or feed [3]. When such invasive species provide economic and culinary benefits to local communities, the cost of their control and eradication increases due to a lack of cooperation and resistance from the local communities. Using gastronomy to control the invasive species population may not be an effective tool, as it could lead to their further commercial breeding and spread into new areas [4,5].

In fact, the catfish has already escaped into several large natural water bodies in India, such as the rivers Ganga, Yamuna, Sutlej and Godavari [6,7]. They are even found in smaller freshwater bodies, such as Lake Periyar in Kerala, which is home to six key endemic fish species of high local and global significance — *Crossochellius periyarensis*, *Lepidopygopsis typus*, *Hypselobarbus periyarensis*, *Garra periyarensis*, *Balitordis Nemacheilus periyarensis* and *N*. *menoni* [8]. Once the catfish escapes into natural environments, its eradication becomes difficult due to its ability to cut through nets, remain undetected and move along the ground from one water body to another. In natural environments, such as lakes and rivers, the catfish aggressively preys on endemic species and has a very high reproduction rate. Their special breathing capability aids them in surviving on land and in shallow muddy waters for prolonged times. In general, invasive species are found to be highly tolerant to degraded environments (such as turbid waters), which gives them an edge over endemic species when competing for the same resources [9,10].

Given the presence of multiple channels through which such exotic species can be introduced into new environments, their early and effective control becomes paramount. In the case of the African Catfish, local communities in South India have not only taken to their commercial breeding, but consumption demand has shifted in their favor. A native fish species, *Clarias batrachus* (locally called *Magur*), which is preferred for consumption, has been reduced to low numbers in its natural habitats due to competition with the African Catfish. Interestingly, given the high similarity between their morphological features and due to the declining availability of *Magur*, some consumers may also have unknowingly switched from *Magur* to the African Catfish over time. One study performed morphological comparisons, using DNA barcode analysis, to test for the extent of substitution between the African Catfish and *Magur* in local markets in India [11]. It was found that up to 99 percent of the locally sold samples were of African Catfish species, even though they were being marketed as *Magur* [11]. In the case of other exotic species, such as the Lionfish in the Caribbean, Asian Carp in Illinois and the Paiche in Bolivia, their consumption has been promoted as a strategy to contain their further population growth and spread. However, it is not clear whether such strategies have been effective [12].

Given the above background, in this paper we ask the question of how to manage the threats posed by the presence of a rapidly spreading exotic fish species in a lake which is also home to several endemic fish species that provide market and non-market benefits to local communities. Further, we address the important issue of a possible behavioral shift in the communities’ consumption patterns toward the exotic species which leads to an increase in their control costs and exacerbates the risk of local extinction of endemic species. While there exists an extensive body of literature applying bioeconomic models for invasive species management (see for example, [13,14]), the aforementioned behavioral aspect has remained largely unexplored.

Based on the insights from the existing literature, the optimal management question related to exotic species (which is, whether to eradicate or control their populations) varies depending on a combination of various ecological, economic and environmental parameters, such as the relative intrinsic growth rates of the species, costs and benefits of control, carrying capacity of the environment, etc. [15]. The nature and costs of control also vary with the life history of the invading species — for those having a high reproduction rate, controlling reproduction is optimal, whereas for those with higher adult survival rate, targeting the adult species becomes optimal [16]. When the invasive fish species of concern provide economic benefits, their optimal stock would need to be kept lower than the maximum economic yield (MEY) level in cases where they adversely impact on the provisioning and supporting services of the surrounding ecosystems. For example, the invasive king crabs provide an annual revenue of 130 million NOK to the community of small-scale fishers in Norway. However, they also damage the ecosystem by reducing commercial fish stocks and adversely impacting on the biodiversity and benthic spices in the region. One study derives the optimal stock level of red crabs while accounting for the negative impact they have on the bycatch costs in the Barents Sea in Norway [17]. It recommends that the optimal stock size for king crabs be kept lower than the MEY level, as this would help with the objective of preserving the ecosystem services of the Barents Sea. Another study [18] provides a case of exotic species management challenge, albeit in the context of exotic plants, where the benefits of preventing the native species from getting crowded out by *Acacia saligna* (an exotic woody plant) need to be balanced against the loss to the livelihoods for those dependent on fuelwood harvesting. It is suggested that it may be economically feasible for the underprivileged communities to switch to wildflower harvesting while the exotic woody plants are targeted through a biological agent.

In regard to managing the catfish, determination of their optimal control efforts would also be affected by society’s willingness to pay for the endemic species threatened by them. The endemic fish species could be valued by the local communities not only for their consumption benefits but for their non-market benefits. If the manager can generate support from local communities through tapping into the non-market valuation of endemic fish species, it would provide additional resources with which to fight the invasion challenge. Further, the non-market value of the endemic fish species could be subject to change owing to a behavioral shift in community preferences. The existing literature on biodiversity valuation points to various factors influencing society’s decisions concerning which species to protect [19]. For example, individuals have been found to give a higher preference to the preservation of at-risk species that are present in their regions but not elsewhere [20]. Some valuation-based studies have measured people’s willingness to pay for prevention programs that reduce the risk of invasion by harmful species [21]. For instance, marine protection programs along the North-Holland beaches are aimed at reducing the threat of arrival of harmful algal bloom species. Using a combination of contingent valuation and travel cost method approaches, it was found that in absence of any prevention measures to control algal blooms, the total loss in welfare to the community would be between €225 and €325 million [21]. Therefore, the non-market value of threatened species provides additional resources with which to guard against the threat of invasive species.

The behavioral response of communities to the presence of invasive species in new environments is another factor that could play a role in determining their long-term survival and spread as well as that of the endemic species threatened by them. It is generally acknowledged that human behavior and economic incentives can influence the level of risks faced by endangered species [22, 23]. If farmers take to commercial breeding of the catfish, the chances of their escaping into natural environments increase. To maximize their incomes, farmers often tend to overstock their ponds, which increases the probability of catfish escaping during rainy seasons when ponds get flooded. While a shift toward the commercial exploitation and demand for catfish is one aspect of the behavioral factors that increases their risk of spread, local fishermen could also change their fishing patterns once the invasive fish species have become established in natural environments such as lakes or rivers. Being heavier, the catfish commands a better price compared to endemic fish species. A change in fishing patterns would raise the opportunity costs of hiring labor needed to control catfish spread. Additionally, there could be intangible costs involved through resistance faced in eradicating an invasive species that supports local livelihoods. If local communities quickly adapt to the invading species, their eradication would become impossible. However, when adaptation responses are endogenous to the stock abundance of the exotic and endemic fish species, the manager of the lake may have more flexibility in deciding whether to eradicate the invasive species or manage their controlled spread where eradication may not be economically feasible.

Despite the significance of community behavioral responses facilitating the spread of invasive species in new environments, this aspect has remained relatively unexplored. In this paper, we address this gap through developing a dynamic optimization model of a lake manager’s decisions relating to exotic fish control when faced with the risk of a future shift in community preferences or a loss in valuation of the endemic fish species. The risk of a shift in the consumption preference toward the exotic fish species is modeled as endogenous and partly controllable by the lake manager. The non-market value of the endemic fish species to the community is also modeled as endogenous. A numerical exercise is performed to derive some key insights.

## Materials and methods

### Model outline

Consider a large lake that serves as a habitat for a representative endemic (or native) fish species. The lake has recently been invaded by the African Catfish. The lake manager faces the task of controlling the spread of this exotic fish species in the freshwater body to protect the endemic variety. A reduction in the stock abundance of endemic fish species will result in a loss of consumption utility to the local community. There is also a non-market value associated with the endemic fish species, as the local community holds a certain willingness to pay toward its preservation efforts.

The catfish preys on the endemic fish species and competes with it for common food resources within the lake. The reproduction rate of the endemic fish species is much lower compared to the catfish, which removes any possibilities of a dynamic equilibrium, whereby both species could co-exist within the lake. The exotic catfish is highly adaptive to new environments and can thrive in degraded water bodies and in shallow muddy conditions. Therefore, when allowed to spread uncontrolled, the catfish easily outcompetes the endemic species, causing their local extinction in the long term. The stock abundance of this predator species, however, is not affected by a reduction in the population of the endemic fish species, as they have multiple hosts within the lake and can prey on small birds or even migrate small distances over land.

Early action by the lake manager could result in an eradication of the exotic fish species, whereas, a delayed response results in their establishment. Once the exotic species population size increases, it causes a significant decline in the stock abundance of the endemic fish species. The local community, owing to a lack of awareness, displays no aversion to the exotic fish species and can easily switch its consumption preferences from the endemic to the exotic variety. Preference switching is more likely to occur when the endemic variety suffers a decline in their stock abundance and there is scarcity of their supply for local consumption. The production and sale of the exotic fish species is made illegal by the local regulator, therefore, if the community were to switch to the exotic fish species, their consumption demands would be met, not through the stock in the lake but through the exotic fish species illegally bred on aquaculture farms. This behavioral response of the community to a reduction in the endemic fish species supply increases the costs of control for the lake manager, as community resistance to their culling increases, as well as the direct costs of hiring labor increase due to higher wages prevailing in catfish aquaculture.

In addition to an increase in control costs, there is a possibility of a loss of non-market value of the endemic stock in the lake. Upon a shift in the community’s consumption patterns, the non-market value of the endemic fish species declines significantly. When faced with such behavioral risks, the lake manager optimizes exotic fish species control efforts in the pre-adaptation scenario to endogenously influence the risk of a community’s behavioral shift. The following section formalizes this idea.

### Model equations

Following the standard bioeconomic modeling approach in fisheries [24], the stock, *x*(*t*), of the invasive fish species evolves as:

$$\dot{x}(t)=\rho_{x}\cdot x(t)\cdot \left(1-\frac{x(t)}{k_{x}}\right)-q_{x}(t),$$ (1)

where *ρ*_*x*_ is its intrinsic growth rate, *k*_*x*_ the carrying capacity, and *q*_*x*_(*t*) the annual catch rate from control (or harvesting) effort *h*_*x*_(*t*) exerted at time *t*. The variable *h*_*x*_(*t*) is the effort that the manager sets to eradicate the exotic fish species, measured in number of hours. Fish yield has been modeled in the literature [25] to exhibit saturation effects with respect to control effort and stock abundance. Accordingly, the relation between catch rate and control effort is given as:

$$q_{x}(t)=h_{x}(t)\cdot l_{x}\cdot \left(\frac{x(t{)}^{h_{1}}}{x(t{)}^{h_{1}}+h_{2}}\right),$$ (2)

where *l*_*x*_ is the maximum possible catch per unit effort, which is adjusted by the term $\left(\frac{x(t{)}^{h_{1}}}{x(t{)}^{h_{1}}+h_{2}}\right)$. Parameters *h*_1_ and *h*_2_ determine how quickly a depletion in the fish stock reduces the effectiveness of catch per unit effort. The dynamics concerning the stock abundance, *y*(*t*), of endemic fish species is given as:

$$\dot{y}(t)=\rho_{y}\cdot y(t)\cdot \left(1-\frac{y(t)+\beta \cdot x(t)}{k_{y}}\right)-q_{y}(t),$$ (3)

where *ρ*_*y*_ is its intrinsic growth rate, *k*_*y*_ the carrying capacity, and *q*_*y*_(*t*) the per-period catch rate. A Lotka-Volterra type competitive interaction effect [26, 27] is incorporated using the *β* parameter, which determines the extent of the adverse impact *x*(*t*) has on $\dot{y}(t)$. Alternative formulations of the competitive interaction processes between the two species could involve relating the carrying capacity of one species to the stock abundance of the competing species (see for example, [24]).

The catch rate of the endemic fish species depends on the harvest effort and the stock of fish species, modeled as:

$$q_{y}(t)=h_{y}(t)\cdot l_{y}\cdot \left(\frac{y(t{)}^{h_{3}}}{y(t{)}^{h_{3}}+h_{4}}\right),$$ (4)

where *h*_*y*_(*t*) is the fish harvesting effort (measured in number of hours spent fishing annually) made by the manager of the lake for deriving economic benefits, and *l*_*y*_ the maximum catch per unit effort that is possible when the stock of endemic fish species is sufficiently large. The term $\left(\frac{y(t{)}^{h_{3}}}{y(t{)}^{h_{3}}+h_{4}}\right)$, which ranges between 0 and 1, adjusts the value of *l*_*y*_ downwards at low levels of endemic stock, where parameters *h*_3_ and *h*_4_ determine how steeply a depletion in the endemic stocks reduces the effectiveness of the catch per unit effort.

The cost, *f*_*y*_(*t*), associated with the harvesting of the endemic species is given as:

$$f_{y}(t)=w_{0}\cdot h_{y}(t{)}^{c_{c0}},$$ (5)

where *w*_0_ is the wage rate (or opportunity cost) per unit of fishing effort and *c*_*c*0_ a parameter that makes the cost increase non-linearly in fishing effort [28]. Likewise, the cost, *f*_*x*_(*t*), associated with controlling the invasive fish species is given as:

$$f_{x}(t)=w_{0}\cdot h_{x}(t{)}^{c_{c1}},$$ (6)

where *c*_*c*1_ is a parameter that makes the control cost non-linear in effort.

The market prices of the two types of fish species are given as *p*_*y*_ and *p*_*x*_. The exotic fish species command a higher price in the local market once the community has adapted to their consumption. We take *p*_*x*_ as being exogenously determined, whereas the price of the endemic species is given as:

$$p_{y}=p_{y0}\cdot {(h_{y}(t)\cdot l_{y}\cdot (\frac{y(t{)}^{h_{3}}}{y(t{)}^{h_{3}}+h_{4}}))}^{-\alpha_{y}},$$ (7)

Where *α*_*y*_ is the price elasticity of demand. To keep the analysis tractable, we do not model demand substitution effects between the various endemic, native and imported categories of fish species that may be traded in the local market. In general, the demand for fish in India has been found to be price sensitive [29].

The local community values the endemic fish species not only for the revenues generated, but for their non-market benefits, *V*_*endem*_(*t*), which is endogenous to the stock of the fish species, given as:

$$V_{endem}(t)=\left(\frac{y(t{)}^{\beta_{0}}}{y(t{)}^{\beta_{0}}+\beta_{1}}\right)\cdot p_{y}(t)\cdot q_{y}(t),$$ (8)

where *β*_0_ and *β*_1_ are parameters governing the steepness with which the endemic value of fish species declines when their stock abundance in the lake gets to low levels. While we model a decline in value with a depletion of stock abundance, it is not unusual to find species (such as rhinos, tigers, and pythons) whose value in the illegal market increases as their stock abundance declines. This phenomenon has been termed as the Anthropogenic Alee Effect [30]. The endemic value of the fish species is difficult to quantify as it could vary significantly based on the type of fish species, elicitation method used, and number of other endemic fish varieties in the local environment. If one infers the revealed non-market value of the endemic fish species from the preservation efforts invested by the communities in regions that face significant threats from exotic invasive fish species, it is usually very low. For the lake manager to invest efforts toward the conservation of the endemic fish species, it is assumed here that the manager can generate, at most, the same amount of additional resources from the local community that is derived through their harvesting for economic benefits. The non-market value of the endemic fish species is modeled here as having an upper limit, which is equal to the current revenues generated through its harvesting. This assumption explains the presence of the term *p*_*y*_(*t*) ⋅ *q*_*y*_(*t*) in Eq (8). It is also worth noting that $\left(\frac{y(t{)}^{\beta_{0}}}{y(t{)}^{\beta_{0}}+\beta_{1}}\right)$ is bounded between 0 and 1. Therefore, when endemic stock abundance is low, the non-market value is significantly lowered even if the revenues generated are high.

The objective of the representative lake manager, in the scenario where the invasive fish species have arrived in the lake but the community has not yet behaviorally adapted to their presence, is to maximize the discounted sum total of per-period utilities that result from the consumption of the endemic fish species harvested from the lake, as well as their non-market benefits:

$$U(x,y)=\max\int_{0}^{\infty }\log\left(p_{y}(t)\cdot q_{y}(t)+(\frac{y(t{)}^{\beta_{0}}}{y(t{)}^{\beta_{0}}+\beta_{1}})\cdot p_{y}(t)\cdot q_{y}(t)-w_{0}\cdot h_{y}(t{)}^{c_{c0}}-w_{0}\cdot h_{x}(t{)}^{c_{c1}}\right)\cdot \exp(-r\cdot t)d\;t.$$ (9)

Eq (9) is maximized subject to exotic and endemic fish stock abundance dynamics. Parameter *r* measures the time preference of the lake manager. Trade in invasive species for consumption purposes is illegal, thus the lake manager derives no positive utility from their harvesting. The first order optimality conditions and their implications are further discussed in Appendix A.

Next, consider that the manager faces the additional challenge of the local community adapting its gastronomical preferences toward the exotic fish species. This adaptation rate could be gradual or sudden, subject to various factors such as the local availability of the exotic fish species, level and effectiveness of regulation, etc. Here, we assume that the probability of adaptation evolves over time and is endogenous to the stock of the exotic fish species in the lake. Specifically, as the stock of the exotic fish species increases (and that of the endemic fish species declines), the probability of the community switching to exotic fish consumption increases. The probability of adaptation is modeled as a hazard function, where the hazard rate, $\dot{\lambda }(t)$, is given as:

$$\dot{\lambda }(t)=\lambda_{0a}\cdot \frac{x(t{)}^{\phi_{0}}}{x(t{)}^{\phi_{0}}+\phi_{1}}.$$ (10)

Parameters *ϕ*_0_ and *ϕ*_1_ determine the rate of change in the hazard rate with an increase in the stock of the exotic fish species, and *λ*_0*a*_ is the maximum possible per-period hazard rate. The tangible effects of adaptation on the lake ecosystem are modeled through an increase in the control costs of the exotic fish species in the post-adaptation scenario. Specifically, the parameter *c*_*c*1_ (as given in Eq 6) increases in value in the post-adaptation scenario.

The value function, *V*_*a*_(*x*,*y*), in the post-adaptation state is arrived at by maximizing:

$$max\;\int_{0}^{\infty }\log\left(\begin{array}{l} p_{y}(t)\cdot h_{y}(t)\cdot l_{y}\cdot (\frac{y(t{)}^{h_{3}}}{y(t{)}^{h_{3}}+h_{4}})+\\ (\frac{y(t{)}^{\beta_{0}}}{y(t{)}^{\beta_{0}}+\beta_{1}})\cdot p_{y}(t)\cdot h_{y}(t)\cdot l_{y}\cdot (\frac{y(t{)}^{h_{3}}}{y(t{)}^{h_{3}}+h_{4}})-w_{0}\cdot h_{y}(t{)}^{c_{a}}-w_{0}\cdot h_{x}(t{)}^{c_{c1}} \end{array}\right)\cdot \exp(-r\cdot t)d\;t.$$ (11)

In the post-adaptation scenario, the stock abundance of the endemic fish species declines significantly, due to an increase in the invasive fish population, and faces local extinction possibilities in the absence of adequate control efforts. In the pre-adaptation scenario, the manager has the opportunity to avoid or postpone the community’s adaptation through keeping the invasive population controlled, as that would promote a healthy endemic population. The pre-adaptation optimization problem is to maximize (see [31] for optimization using hazard functions):

$$\max\;\int_{0}^{\infty }(\begin{array}{l} \log\left(\begin{array}{c} p_{y}(t)\cdot h_{y}(t)\cdot l_{y}\cdot (\frac{y(t{)}^{h_{3}}}{y(t{)}^{h_{3}}+h_{4}})+(\frac{y(t{)}^{\beta_{0}}}{y(t{)}^{\beta_{0}}+\beta_{1}})\cdot p_{y}(t)\cdot h_{y}(t)\cdot l_{y}\cdot (\frac{y(t{)}^{h_{3}}}{y(t{)}^{h_{3}}+h_{4}})\\ -w_{0}\cdot h_{y}(t{)}^{c_{c0}}-w_{0}\cdot h_{x}(t{)}^{c_{c1}} \end{array}\right)\exp(-r\cdot t)\cdot \exp(-\lambda (t))\\ +V_{a}(x,y)\cdot \exp(-r\cdot t)\cdot \exp(-\lambda (t))\cdot \lambda_{0a}\cdot \frac{x(t{)}^{\phi_{0}}}{x(t{)}^{\phi_{0}}+\phi_{1}} \end{array})dt,$$ (12)

subject to the endemic and the invasive fish population dynamics and the rate of change of the hazard rate. The first order optimality conditions are presented in Appendix A. In the next section, we perform a numerical exercise to derive some key insights from the above model.

### Parameter calibration

The numerical example mimics the case of a large lake in South India, where the invasive African Catfish are spreading rapidly, threatening pristine habitats and causing local extinction of several endemic species. Calibrated parameter values are presented in Table A in S1 Appendix. The African Catfish has a high reproduction rate and the number of eggs produced per kg of weight of an adult fish can be as high as 150,000 [32]. In the absence of any control efforts, the catfish can quickly take over a new environment. To incorporate this possibility, an intrinsic growth rate of 0.5 is selected, which implies that, in a large lake, the catfish can grow from a population of 1,000 to 30,000 in about 10 years’ time. The area of the lake is assumed to be roughly 30 km^2^ (3,000 ha). The lake has a carrying capacity of 65,000 fish species, calculated using a catfish density of 21.76 fish species/ha [33].

The value of the parameter *l*_*y*_, measuring maximum catch per unit effort (in Eq 4), is taken to be 0.6 fish per hour, based on catch coefficient of similar fish species in River Cauvery in the Western Ghats in India [34]. The representative endemic variety has a lower intrinsic growth rate of 0.1. At this growth rate, when left undisturbed, a starting stock of 20,000 would double in size to 40,000 in about 15 years. Though it would take the same initial stock size more than 50 years to reach the full carrying capacity level of 65,000. These assumptions also imply that when the invasive catfish have arrived in the lake (with a starting population of 1,000), the initial stock of 20,000 endemic fish species will be reduced to 10,000 in about 20 years (in the absence of harvesting for human consumption). Similarly, when the starting population of both fish types is 20,000, the endemic fish species population will be reduced by half in only 10 years.

The costs of catching the endemic and invasive fish types are estimated based on a wage rate of 0.3 USD per hour (or 2.4 USD per day, roughly equivalent to 144 Indian National Rupees (INR) per day). However, as the number of fishing hours increases, the labor costs increase non-linearly. If the local community adapts to the presence of the invasive fish species in the natural environment, resulting in a shift in the consumption demand from endemic to invasive fish variety, the costs of controlling the catfish species in the lake increase. This is due to an increase in the opportunity cost of labor, which reflects the higher revenues generated through aquaculture of the catfish. The minimum catfish price in the local market is 5 USD (roughly 300 INR) [2], whereas the price of the endemic fish species is only 2.5 USD. These prices can also vary with the fish species size, demand and supply conditions. The African Catfish can, in fact, reach weights of up to 60 kg [35].

Finally, the hazard rate of adaptation is assumed as a function of the stock of invasive fish species in the lake. As there is a direct one-to-one relation between a higher stock of the exotic fish species and lower stock of the endemic species, an increase in the stock of catfish would lower the stock of endemic fish species over time, thereby contributing to a shift in the consumption demand away from the endemic fish species and in favor of the catfish. The hazard function is calibrated such that if *x*_0_ = 10,000 and *y*_0_ = 20,000, the probability of community adaptation in eight years, increases to more than 50 percent.

**Fig 1:**
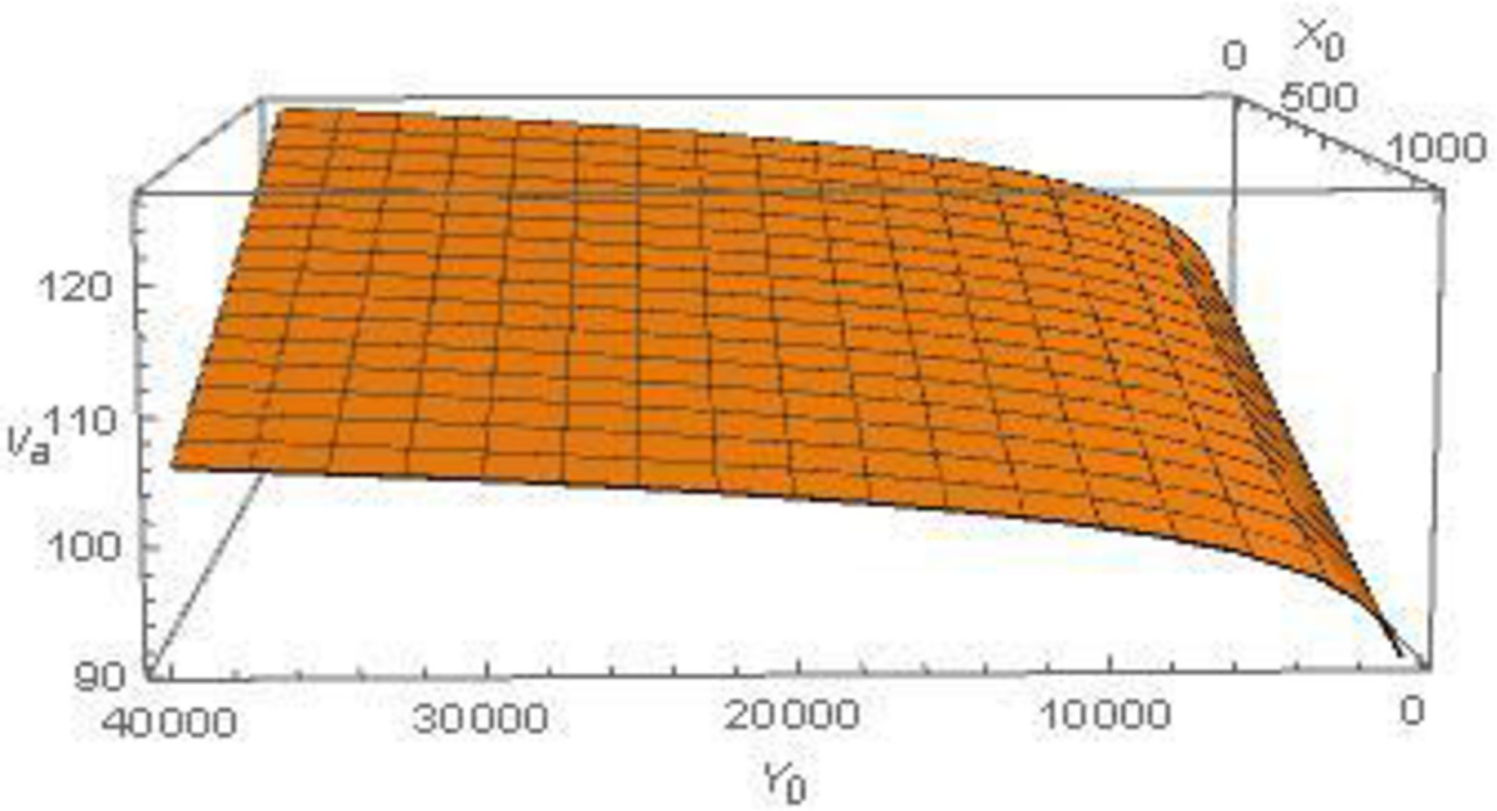
Post-adaptation value as a function of the initial stocks of endemic and exotic fish species. *x*_0_ = initial population level of the invasive fish; *y*_0_ = initial population level of the endemic fish; *V*_*a*_ = post-adaptation value function.

The numerical example is solved using the general algebraic modeling system (GAMS) software with an *minlp* solver option. The time horizon is taken as 100 years, which for a discount rate of five percent mimics an infinite horizon problem. The post-adaptation value function is solved through repeatedly optimizing the lake manager’s post-adaptation utility for various starting levels of stocks of the invasive and endemic fish species. The resulting value function is calibrated using a non-linear regression estimator in *stata* software. The calibrated value functions are presented in Table A in S1 Appendix for two scenarios. The first (Eq 11 in Table A in S1 Appendix) involves a higher cost of adaptation, and the second incorporates a higher cost of adaptation along with a complete loss of the endemic value. Fig 1 presents a plot of the calibrated value function for the post-adaptation scenario when only the cost of control increases (and endemic value is not lost).

## Results

The purpose of the numerical exercise is to generate insights related to the impact of key parameters on the long-term distribution of the endemic and invasive species in the lake environment. Specifically, we are interested in exploring how a variation in the cost of controlling the exotic species affects optimal strategies. It is worth reiterating here that optimality in this paper is being referred to from the lake manager’s perspective and is guided by the choice of his utility function. The cost parameter of interest is *c*_*c*1_, which is varied between 1.2 and 2. A value of 2 would make the cost of control highly non-linear, thereby creating significant tradeoffs between current eradication efforts spent on invasive species control and future values derived from the endemic fish species. As the inter-species dynamics is affected by the initial stocks of the exotic and endemic species, we also vary these parameters to derive additional insights. The initial population of the endemic fish species (*y*_0_) in a healthy lake environment would be close to its carrying capacity level of 65,000, whereas, the same would be much lower (e.g., 10,000) in a significantly overexploited lake. The initial population of the invasive fish species, *x*_0_, could range between just a few fish and a population as large as 10,000. Initial stock of invasive species, in particular, is a key determinant of their future establishment potential in new environments. Specially, fast-growing invasive fish species, if not detected during the initial stages of their introduction, can quickly multiply and takeover a lake environment. We further explore the implications of variations in the reproduction rate (*ρ*_*y*_) of the endemic species, as this affects their survival chances when threatened by an aggressive predator. The reproduction rate is varied from 0.1 to 0.5, where the upper value matches the reproduction rate of the catfish. Finally, we consider a few scenarios where the risk of behavioral adaptation comes into play. The parametrization of the risk of adaptation ($\dot{\lambda }$) is provided in Table A in S1 Appendix.

**Fig 2:**
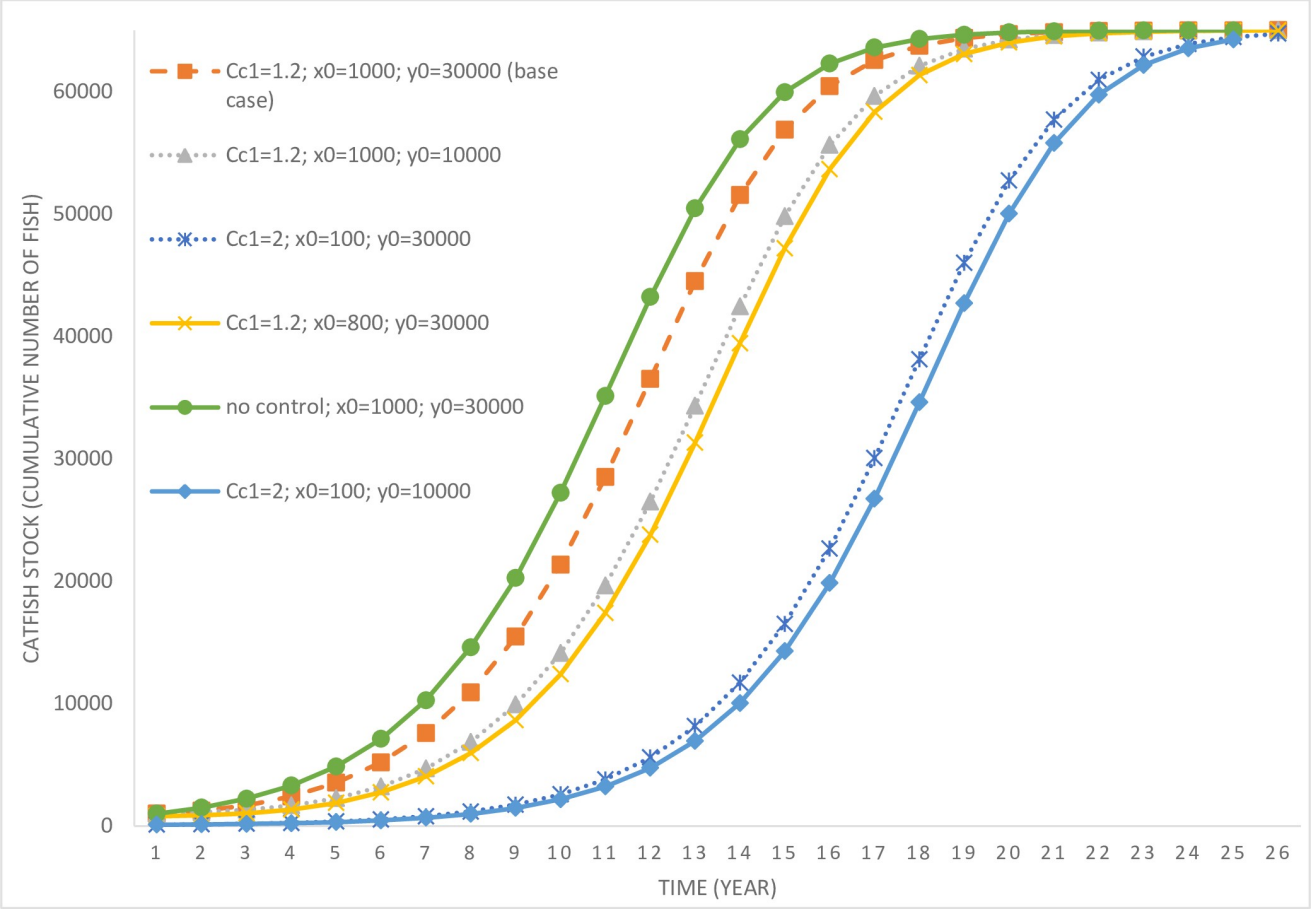
Time paths of catfish population under various scenarios involving variations in the initial size of invading and endemic fish species stocks and costs of control. *c*_*c*1_ = parameter determining the cost of invasive species control; *x*_0_ = initial population level of the invasive fish; *y*_0_ = initial population level of the endemic fish; ‘no control’ refers to the scenario where the lake manager exerts no efforts toward reducing exotic fish species stock.

**Fig 3:**
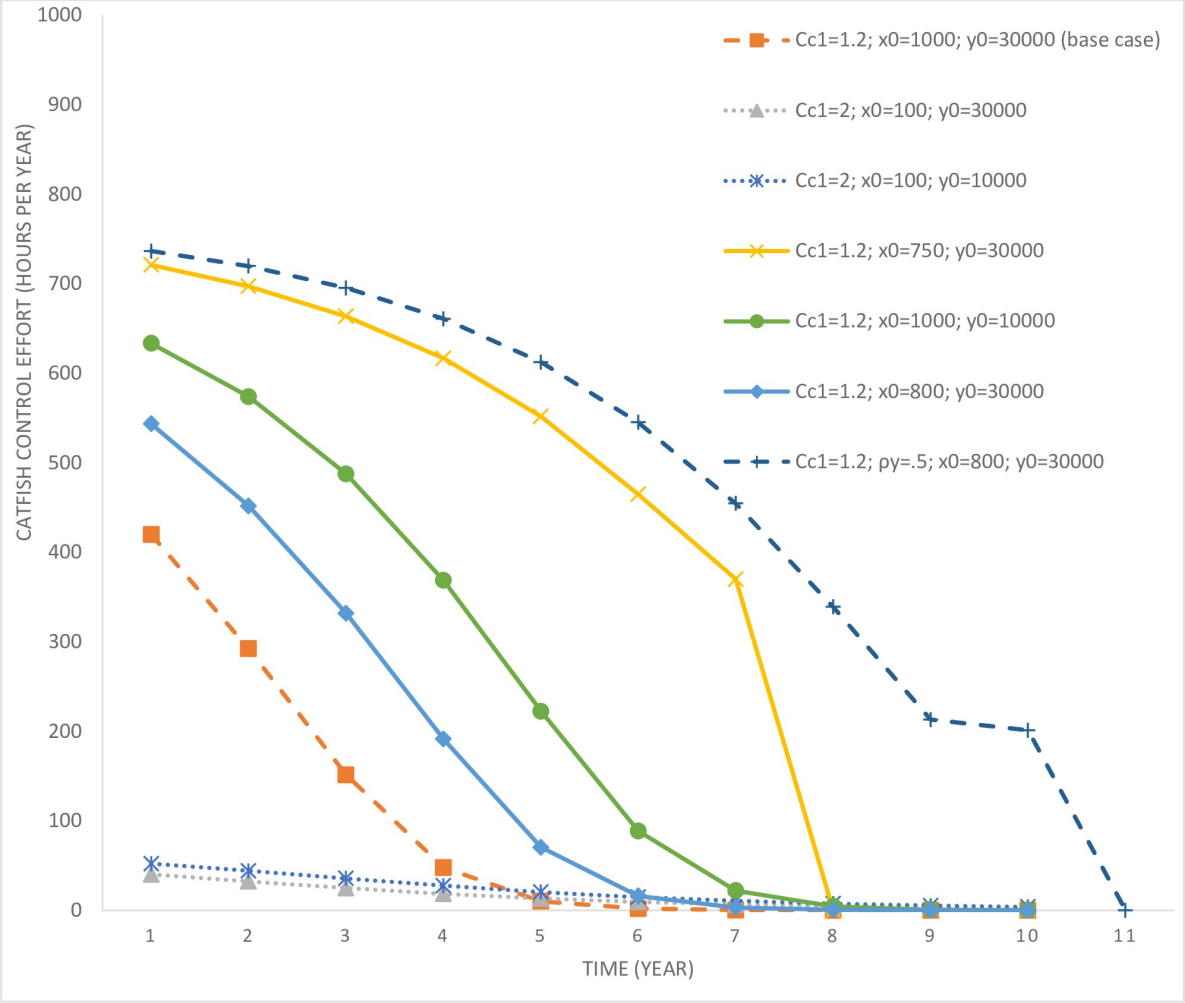
Time paths of catfish control efforts under scenarios involving variations in initial invading fish species stock and the costs of control. *c*_*c*1_ = parameter determining the cost of invasive species control; *x*_0_ = initial population level of the invasive fish; *y*_0_ = initial population level of the endemic fish; *ρ*_*y*_ = intrinsic growth rate of the endemic fish species.

The base case (and its variations) assumes that the invasive fish species is already present in the lake, however, its impact on the endemic fish population is small enough not to cause any changes to the community’s gastronomical preferences. The community continues to consume the endemic fish species and has not yet adapted to the presence of the catfish in their environment. In the base case scenario, the initial stock of the catfish is 1,000 and the endemic fish population is 30,000 (*c*_*c*1_ = 1.2; *x*_0_ = 1,000; *y*_0_ = 30,000). In this scenario, the optimal response of the lake manager is to invest in a small level of control effort and delay the time to complete extinction of the endemic fish species. This is evident from Fig 2, which depicts the stock of catfish over time, which reaches close to its carrying capacity by year 20. The level of control effort (in number of hours per year) is depicted in Fig 3.

**Fig 4:**
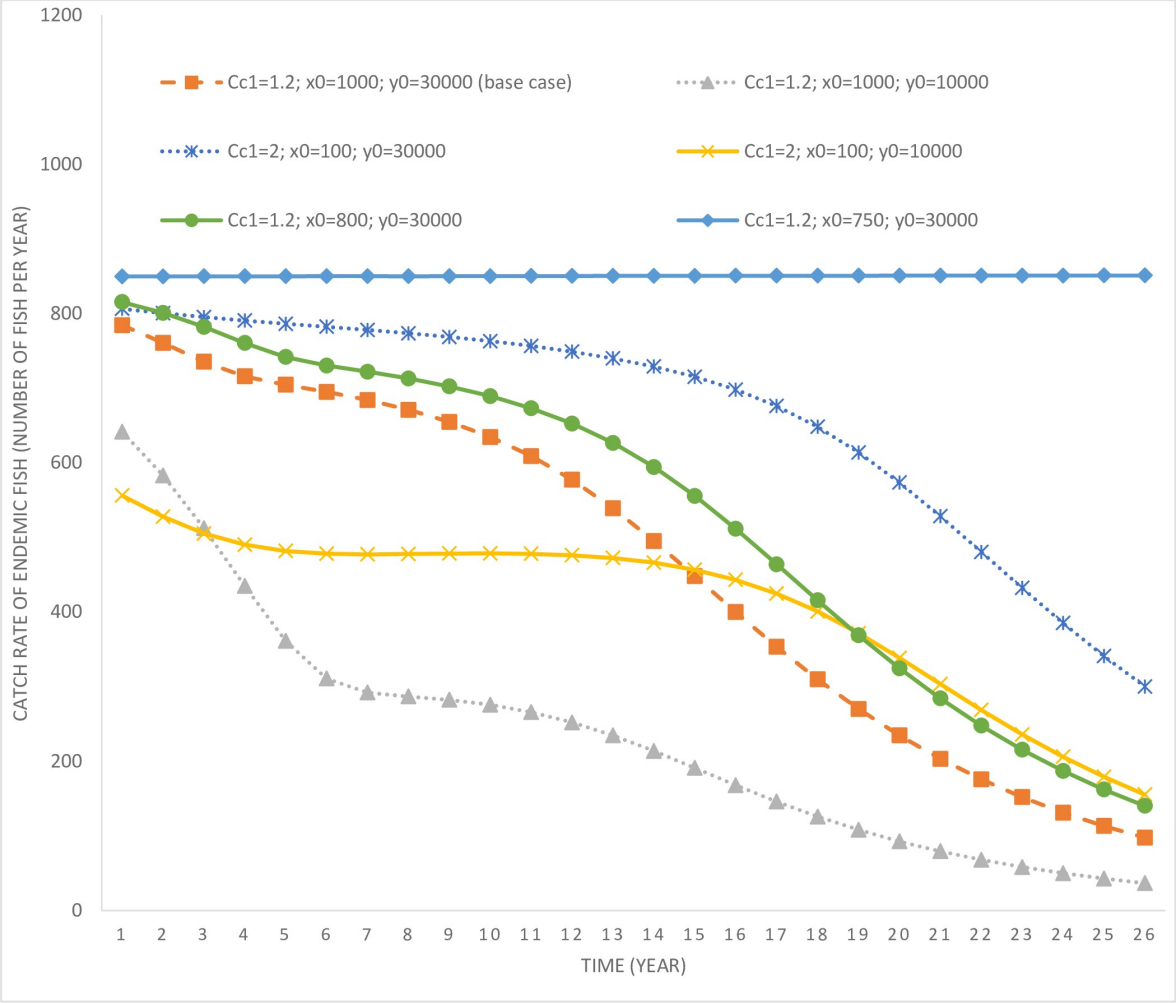
Time paths of harvesting of endemic fish species populations. *c*_*c*1_ = parameter determining the cost of invasive species control; *x*_0_ = initial population level of the invasive fish; *y*_0_ = initial population level of the endemic fish species.

**Fig 5:**
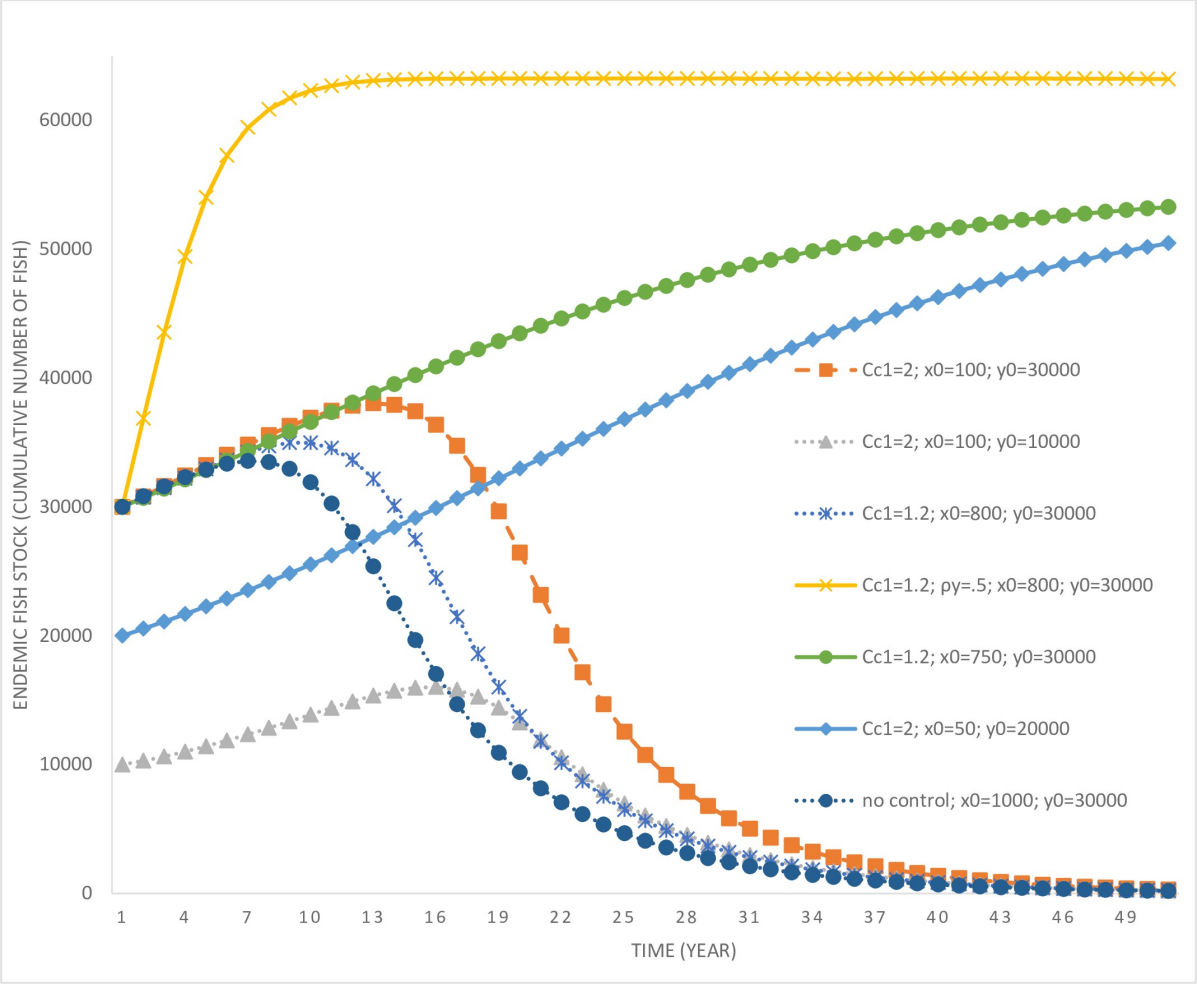
Endemic fish species populations over time under various scenarios involving variations in the initial invading stock and costs of control. *c*_*c*1_ = parameter determining the cost of invasive species control; *x*_0_ = initial population level of the invasive fish; *y*_0_ = initial population level of the endemic fish; ‘no control’ refers to the scenario where the lake manager does not exert any effort toward reducing exotic fish species stock.

It is only after year 10 that the catfish population starts to increase rapidly (as the control effort drops to zero past that time). While the manager delays the rapid multiplication of the catfish population in the lake, their eradication is not optimal under this scenario. This is due to the high level of initial catfish population stock that would require significant expenditures for eradication. Optimal control effort is also affected by the stock of the endemic fish species in the lake. Consider another scenario (*c*_*c*1_ = 1.2; *x*_0_ = 1,000; *y*_0_ = 10,000), where the invading stock is high at 1,000, but the endemic stock is much lower at 10,000 (in comparison to the previous scenario). In this scenario, the endemic population is more vulnerable to local extinction given its low stock size. Under this scenario, control effort is much higher compared to the base case, which allows more time for facilitating endemic fish population growth. The harvesting rate of endemic fish species in this scenario is also relatively lower compared to the base case scenario (see Fig 4), which further assists with their rapid population growth (see Fig 5).

Consider another scenario (*c*_*c*1_ = 1.2; *x*_0_ = 800; *y*_0_ = 30,000) where the initial invading population is marginally lower at 800 and the endemic population higher at 30,000. In this scenario, the control effort aimed at curbing catfish growth is marginally higher compared to the base case, however, the harvesting rate of the endemic fish species is also higher. Higher harvesting of the endemic population is a result of its higher initial population and the cost effectiveness of harvesting effort at higher populations. However, even under this scenario, it is not optimal to eradicate the catfish population given its high initial stock. Therefore, based on the above three scenarios, we can draw the following inferences.

### Summary of results 1

For a given cost of invasive species control and initial population size, control effort increases when the initial level of endemic population is lower compared to when it is higher. Similarly, for a given cost of invasive species control and initial endemic population level, control effort increases when the initial invading population level is lower compared to when it is higher.

The last result may appear counterintuitive, that one would exert more control effort at a lower invading population compared to when the population size is higher. However, there are more benefits of control at lower population levels as it delays the future spread of the exotic fish species. Also, the cost of control is lower, as lower numbers of fish species need to be culled. Further, it may be economical to eradicate the invasive fish species at lower populations compared to when their population is higher. This intuition will be verified later, but first we explore the implications of variations in the cost of control. A scenario where the local community has already gastronomically adapted to catfish invasion leads to an increase in its control costs. When *c*_*c*1_ = 2; *x*_0_ = 100; *y*_0_ = 30,000, the initial invading population (in the lake) is much lower, but the cost of control has increased significantly. In this scenario, the manager delays the growth in catfish population by exerting some level of control effort but does not find eradication to be an optimal strategy. Given the low level of initial population and the control effort, under this scenario it takes a long time for the catfish population to reach its carrying capacity and replace the endemic variety. However, the longest time taken is for a scenario where *c*_*c*1_ = 2; *x*_0_ = 100; *y*_0_ = 10,000, which requires a marginally higher control effort compared to the previous case due to the vulnerability of the endemic fish species owing to their low initial stock.

Thus far, we have established that under scenarios where it is optimal to delay the growth in catfish populations instead of eradication, a more vulnerable endemic population requires a higher control effort. However, all the above scenarios lead to a local extinction of the endemic fish species in the long run and result in the lake being overtaken entirely by the catfish. This occurs either due to a higher initial size of the invading catfish population, or a higher cost of their control, or a combination of the two factors.

**Fig 6:**
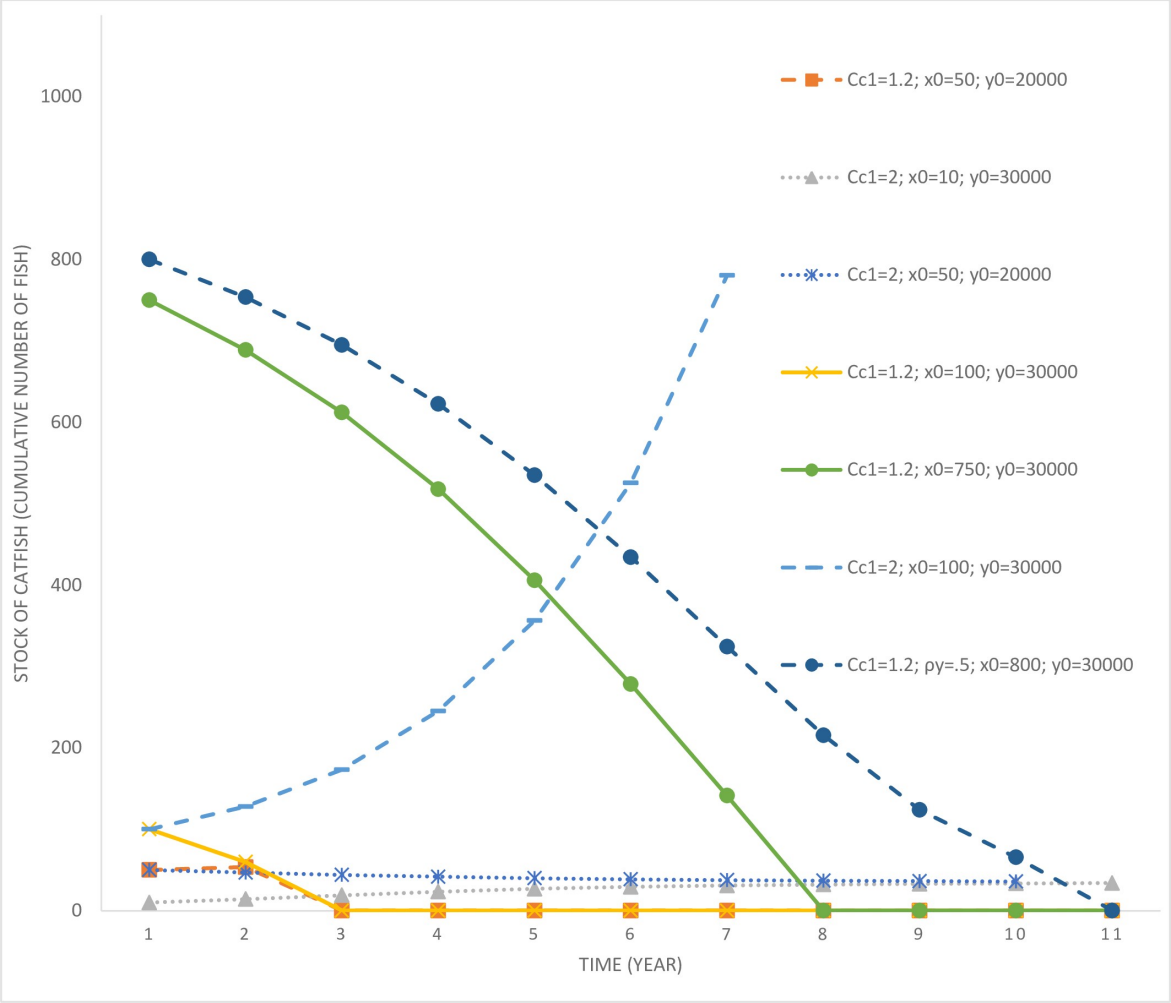
Scenarios where it is optimal to eradicate catfish populations compared with scenarios where the population is allowed to increase over time. *c*_*c*1_ = parameter determining the cost of invasive species control; *x*_0_ = initial population level of the invasive fish; *y*_0_ = initial population level of the endemic fish; *ρ*_*y*_ = intrinsic growth rate of the endemic fish species.

Next, we consider some scenarios where the manager may find eradication of the catfish optimal. Previously, it was observed that in the scenario *c*_*c*1_ = 1.2; *x*_0_ = 800; *y*_0_ = 30,000, eradication of the catfish was not an optimal strategy. In a new scenario, where the initial invading catfish population size is reduced to 750 (see Fig 6, scenario *c*_*c*1_ = 1.2; *x*_0_ = 750; *y*_0_ = 30,000), optimal control results in declining catfish population and their decimation in the long run.

This scenario highlights the possibility of existence of a threshold initial stock level (keeping other parameters unchanged), below which it becomes optimal to switch from partial control to eradication. This switching stock level is affected by various factors such as the cost of control, level of the endemic fish species stock abundance, reproduction rates, etc. For example, consider another scenario, *c*_*c*1_ = 1.2; *ρ*_*y*_ = 0.5; *x*_0_ = 800; *y*_0_ = 30,000, where the reproduction rate of the endemic fish species is equal to that of the invasive fish species. Previously, under the scenario *c*_*c*1_ = 1.2; *x*_0_ = 800; *y*_0_ = 30,000, it was observed that given the initial stock size of 800 for the invading fish species, it was not optimal for the manager to go for eradication. However, under a higher reproduction rate, eradication becomes an optimal strategy. As the endemic fish species can now grow faster, it mitigates the crowding out influence that the invasive fish species have on their stock abundance. A better endemic stock abundance justifies selecting a higher control effort and achieving eradication. Whereas, when the endemic fish species have a lower reproduction rate, a similar level of control effort becomes uneconomical. The endemic fish population is significantly reduced by the time the invasive fish species is eradicated, and the discounted values of subsequent revenues and non-market benefits are too low.

**Fig 7:**
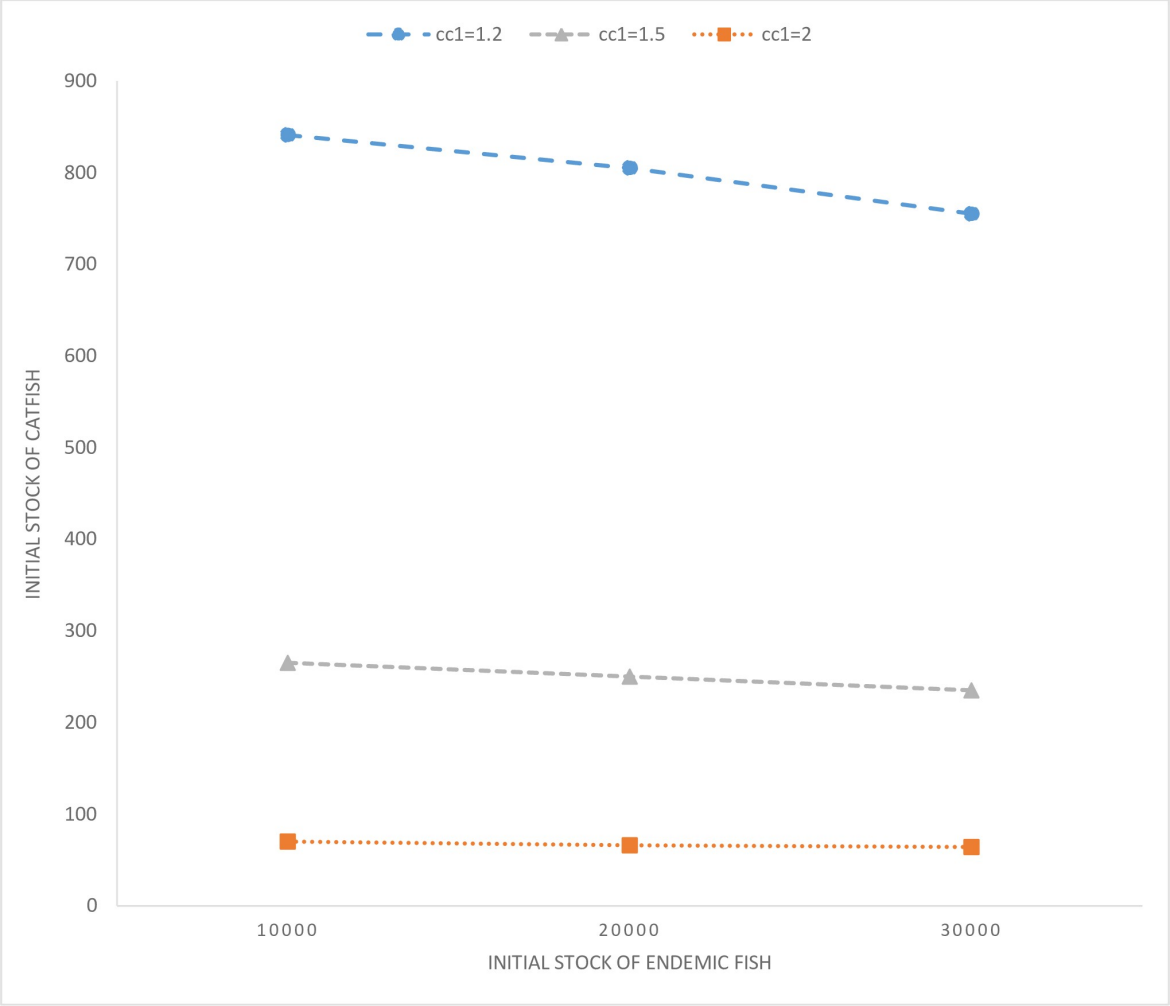
Threshold levels of starting catfish populations, above which eradication is not optimal, depicted for varying costs of control.

We also explore the role of cost of control in influencing a switch in the optimal control policy. Compare scenarios *c*_*c*1_ = 2; *x*_0_ = 100; *y*_0_ = 30,000 and *c*_*c*1_ = 1.2; *x*_0_ = 100; *y*_0_ = 30,000. In the case with higher control costs, the catfish population increases over time (complete time path is not depicted in Fig 6 for ease of comparison), whereas, under lower costs, it is optimal to go for eradication. Further, eradication is also optimal when the invading population size is much lower at 10 or 50 individuals, even when the cost of control is higher. We also derive threshold levels (beyond which eradication is not optimal) of catfish population as a function of the initial endemic stock abundance for three different control cost scenarios (see Fig 7). It is evident that a lower level of endemic fish species stock increases the threshold, however, an increase in control costs, *ceteris paribus*, lowers the same thresholds. Based on these results, we can summarize key insights.

### Summary of results 2

For a given initial population level of the endemic species, as the initial population of the invading fish species is increased, the optimal control strategy switches from eradicating the invasive fish species to controlling their spread over time. Similarly, for a given combination of initial population sizes of the endemic and invasive fish species, a lower cost of controlling the exotic fish species makes their eradication optimal, whereas, at higher costs, the strategy switches to delaying the time to extinction of the endemic fish species.

**Fig 8:**
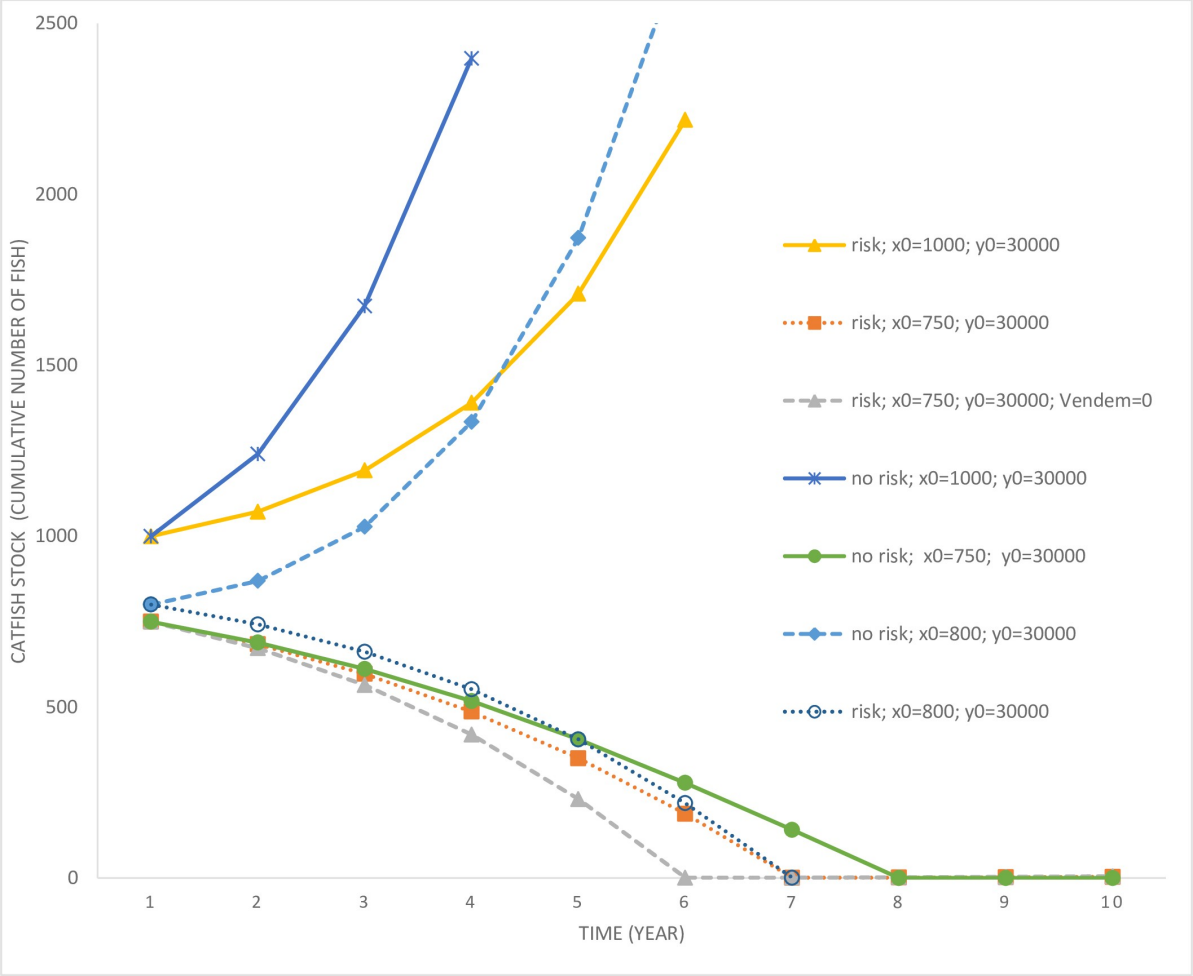
Catfish populations over time under adaptation risk scenarios. ‘risk’ refers to scenarios that incorporate the behavioral adaptation risk, whereas ‘no risk’ refers to scenarios that exclude such risks. ‘Vendem = 0’ refers to a scenario where the non-market value of endemic fish species reduces to zero in the post-adaptation stage.

Finally, we consider some scenarios where the invasive species have arrived and the risk of community adaptation is present. If the invasive fish stock abundance increases to high levels, the risk of community adaptation increases significantly. The result of adaptation is an increase in the cost of control for the lake manager. Fig 8 depicts catfish populations for scenarios involving the risk of adaptation.

**Fig 9:**
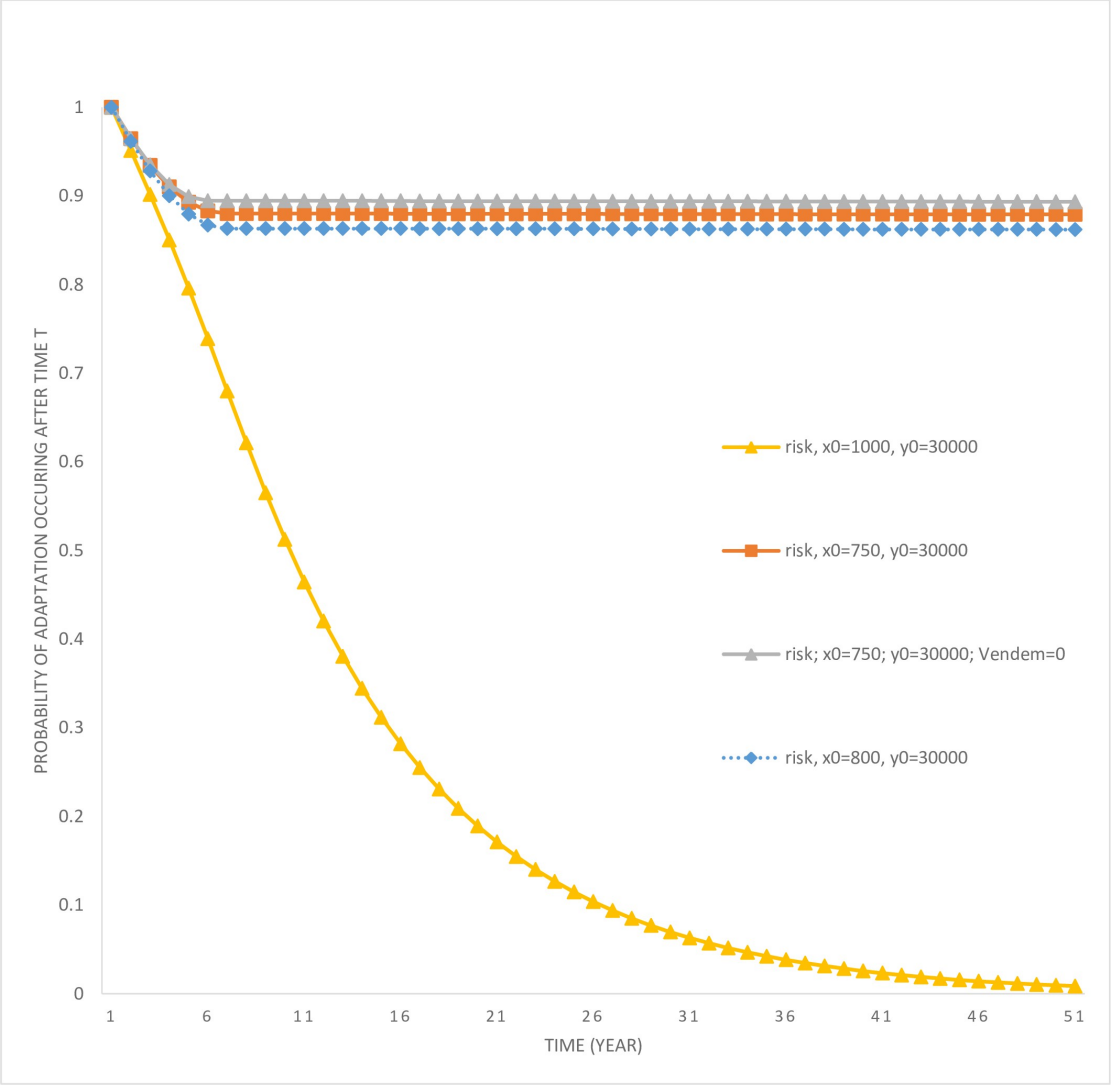
Survival probability of community adaptation to catfish invasion. The survival probability is given by the term *exp*(−*λ*(*t*)), and measures the probability that the adaptation has not occurred until time *t*.

In the presence of risk, the catfish population is controlled to keep its stock lower compared to a scenario where the risk does not exist (compare scenarios risk; *x*_0_ = 1,000; *y*_0_ = 30,000, and no risk; *x*_0_ = 1,000; *y*_0_ = 30,000). This should be intuitive, as through controlling the catfish population the risk of adaptation can be kept lower. The survival probability (see Fig 9) is above 10 percent even after 25 years.

Another scenario, where *x*_0_ = 750; *y*_0_ = 30,000, leads to a much more rapid eradication of the catfish population when in the presence of risk compared to when risk is non-existent. This result is similar to the previous case, except that in this case it is optimal for the manager to eradicate the catfish from the lake. Next, when the manager faces the possibility of a loss in the non-market value of the endemic fish species with a community adaptation to catfish consumption (scenario risk; *x*_0_ = 750; *y*_0_ = 30,000; *V*_*endem*_ = 0’), this leads to even an faster rate of eradication. Consequently, endemic fish species survival rate under this scenario is the highest of all three risk scenarios considered so far. Finally, consider the threshold level exotic fish species stock case discussed earlier which led to a switch in the control strategy (compare scenarios no risk; *x*_0_ = 800; *y*_0_ = 30,000, and risk; *x*_0_ = 800; *y*_0_ = 30,000). In the no-risk case, optimal control leads to an increase in the exotic fish species stock, whereas under the risk scenario they are eliminated. Therefore, risk of adaptation also influences the threshold level associated with control strategies. We summarize these results below.

### Summary of results 3

The presence of a risk of community behavioral adaptation to the invasive fish species results in higher levels of control efforts compared to a scenario where no such risk is present. The threshold level of exotic fish species stock abundance at which eradication becomes optimal is influenced by the risk of gastronomical adaptation.

## Discussion

The model presented in this study abstracts away from the real-world situation in a number of ways. Ecosystems, such as large lakes, can be simultaneously invaded by multiple species, thereby increasing the complexity of management challenges. For example, the common carp (*Cyprinus carpio*) is another invasive species with a high reproduction rate and rapid spreading capability that is considered a big threat in the study area. Common carp has already been found in the Ganga river [36]. When common carp and catfish together invade a lake, it would not only reduce the endemic fish species diversity but affect the lake’s environment through increasing bioturbation and sediment erosion, competing with water fowl for food, and lowering aquatic invertebrate and plant populations [37]. In the inland freshwaters of Northern Italy, it was found that a combination of exotic fish species (including wels catfish, common carp and crucian carp) had completely replaced the native species in a couple of xenodiversity hotspot regions [38]. Similarly, there may exist synergistic effects between the exotic fish species and environmental and geomorphological processes which could adversely impact on the distribution of the native species and even drive their extinction [39]. For example, exotic species, in comparison to native species, not only have a higher reproduction rate but are far more capable of surviving in degraded environments [40]. In a study conducted on 91 late-invasion-stage rivers in Northern Italy, it was found that the combined effects of eutrophication and exotic species (such as common carp, crucian carp and common bream) had pushed the native species close to extinction [39]. Further, exotic species were more likely to be nested at lower altitudes where the eutrophication level was higher, in comparison to native species which were forced to migrate to high-altitude areas.

The study area, in the context of the model presented in this paper, mirrors the aforementioned threats and complexities. Lake water ecosystems across India suffer from high levels of eutrophication due to industrial, urban and agricultural runoffs [41]. This study does not explicitly model the adverse impacts of eutrophication on species diversity, even though eutrophication by itself can significantly reduce species richness in waterbodies [42]. Effectively controlling exotic species in degraded lake environments may require additional measures such as biomanipulation, where planktivorous fish species are introduced to reduce the algae present in lakes [42]. This may further increase the costs of invasive species control. Examples of alternative measures, such as creation of watershed areas to filter nutrients flowing into lakes or use of water treatment plants, exist globally, however, such options are not cheap [43]. For example, just to reduce the nitrate levels in Lake Bloomington, Illinois would cost upwards of 10 million USD (for a 20 million gallon per day capacity plant). It is unlikely that local communities in India would be willing to pay such large sums to preserve endemic species in lakes.

Further, the nature of regulatory mechanisms governing the use of lakes for fishing also influences the success of exotic fish species–related intervention measures. In this study we assumed that a manager can control exotic fish species in the lake without much outside intervention. However, controlling catfish spread in open-access fisheries would pose additional sets of challenges. In the presence of fishing permits or quotas, illegal and unreported harvesting of endemic (or exotic) fish species would alter the inter-species dynamics and introduce uncertainty in the management process. This would become especially problematic at low levels of endemic stock when fish species are more vulnerable to extinction. Similarly, community preferences for exotic fish species may lead to a resistance against eradication efforts. Finally, illegal aquaculture of catfish in private ponds by local communities could also undermine a lake manager’s control efforts, as catfish can easily migrate from private farms to lake ecosystems during the monsoon season when flooding and overflowing of water bodies is common. Therefore, pest eradication efforts would become very costly and prone to failure under such situations. As aquaculture of catfish on private farms is economically rewarding, cooperation from local communities would become difficult to obtain. The model presented in this paper and its findings should be considered in the light of these limitations and challenges.

## Conclusion

When invasive species, possessing superior ability to survive and propagate, invade a new environment, they threaten the survival of several endemic species. The challenge of conserving such at-risk endemic species can be further aggravated due to local communities’ behavioral responses, which could range from a low willingness to preserve native biodiversity to switching their consumption preferences in favor of the exotic species. A shift in gastronomical preferences of local communities toward the exotic species increases the costs of their eradication and contributes to a loss in the value of the endemic species.

In this paper, we addressed this behavioral challenge associated with exotic species management using the example of the African Catfish invasion in India. Several key management insights emerged from the analysis. When faced with the situation where the catfish are already present in the lake, the manager may not find eradication optimal if the costs of their control are high or when their initial population is high. This results in an eventual takeover of the lake by the invading species and causes a local extinction of the endemic species. The initial stock of the endemic species and its valuation by the community also become key factors when determining optimal control effort levels. When the endemic stock is lower, a higher control of the exotic catfish population (combined with a lower harvesting of the endemic fish) is required to allow endemic stock abundance to improve over time. There exists a threshold level of initial stock of the exotic population, below which it is optimal to invest in eradication and above which eradication is not optimal. This threshold stock level is influenced by several factors including the reproduction rates of the endemic and exotic species, the costs of eradication and the risk of gastronomical adaptation. When the initial population size of the exotic fish species is below a level that makes eradication optimal, a high level of harvesting of endemic species for consumption benefits becomes possible. However, this is an ideal scenario which is unlikely to manifest in the real world where detection of the invasive fish species in pristine environments is often made after it has become well established.

When the manager faces an endogenous risk of the community adapting to the exotic species, the optimal response is to make higher control efforts in the presence of the risk compared to when the risk is absent. However, the costs of control and initial stock of the invading population still play a role in determining whether to allow a controlled rate of spread or opt for eradication. The worst-case scenario, which leads to an increase in the cost of control in the post-adaptation world and a loss in the non-market value of the endemic species, requires the highest level of control efforts in the pre-adaptation phase. Our results generated the important insight that when the invasive fish species population is close to behavioral change thresholds, a higher level of control effort can make a significant difference to the future distribution of species in the environment.

From a policy perceptive, having advance knowledge of the control costs associated with eradication and the costs of a long-term establishment of the exotic species is crucial. If there is a clear local awareness of the costs of complete eradication along with key parameters, such as the level of stock beyond which control costs become very high or the level of endemic fish stock below which a behavioral shift is imminent, it would help influence society’s decisions with respect to contributing additional resources toward invasive species control. To prevent the spread of invasive species in new environments and control the extent of behavioral adaptation in communities where the exotic species have not yet arrived, the non-market value of endangered endemic species also plays an important part. It is likely that an improvement in the willingness to conserve endemic species will also impact on a community’s preferences toward exotic species consumption. Therefore, generating higher awareness of the non-market values of endemic species could help with the effort to control the establishment of exotic pests in new environments.

## Appendix A: Current value Hamiltonian and first order conditions

The current value Hamiltonian of the optimization problem in Eq (9) can be written in expanded form as:

$$\begin{array}{l} \;\log\left(\begin{array}{c} \underset{revenue\cdot from\cdot endemic\cdot fish}{\underset{︸}{\left(p_{y0}\cdot {\left(h_{y}(t)\cdot l_{y}\cdot (\frac{y(t{)}^{h_{3}}}{y(t{)}^{h_{3}}+h_{4}})\right)}^{-\alpha_{y}})\cdot h_{y}(t)\cdot l_{y}\cdot (\frac{y(t{)}^{h_{3}}}{y(t{)}^{h_{3}}+h_{4}}\right)}}+\\ \underset{non-market\cdot value\cdot of\cdot endemic\cdot fish}{\underset{︸}{\left(\frac{y(t{)}^{\beta_{0}}}{y(t{)}^{\beta_{0}}+\beta_{1}})\cdot (p_{y0}\cdot {\left(h_{y}(t)\cdot l_{y}\cdot (\frac{y(t{)}^{h_{3}}}{y(t{)}^{h_{3}}+h_{4}})\right)}^{-\alpha_{y}})\cdot h_{y}(t)\cdot l_{y}\cdot (\frac{y(t{)}^{h_{3}}}{y(t{)}^{h_{3}}+h_{4}}\right)}}\\ \underset{\cos t\cdot of\cdot harvesting\cdot endemic\cdot fish}{\underset{︸}{-w_{0}\cdot h_{y}(t{)}^{c_{c0}}}}\;\underset{\cos t\cdot of\cdot harvesting\cdot catfish}{\underset{︸}{-w_{0}\cdot h_{x}(t{)}^{c_{c1}}}} \end{array}\right)+\\ \mu_{x}\cdot \left(\rho_{x}\cdot x\left(t\right)\cdot \left(1-\frac{x\left(t\right)}{k_{x}}\right)-\left(h_{x}\left(t\right)\cdot l_{x}\cdot \left(\frac{x{\left(t\right)}^{h_{1}}}{x{\left(t\right)}^{h_{1}}+h_{2}}\right)\right)\right)+\mu_{y}\left(\rho_{y}\cdot y\left(t\right)\cdot \left(1-\frac{y\left(t\right)+\beta \cdot x\left(t\right)}{k_{y}}\right)-\left(h_{y}\left(t\right)\cdot l_{y}\cdot \left(\frac{y{\left(t\right)}^{h_{3}}}{y{\left(t\right)}^{h_{3}}+h_{4}}\right)\right)\right). \end{array}$$ (A1)

In Eq (A1), *μ*_*x*_ and *μ*_*y*_ are the shadow prices of the stocks of the exotic and the endemic fish species, respectively. The first order condition with respect to invasive species control effort, *h*_*x*_(*t*), is given as:

$$\frac{-c_{c1}\cdot w_{0}\cdot h_{x}(t{)}^{c_{c1}-1}}{\left(\begin{array}{c} \underset{revenue\cdot from\cdot endemic\cdot fish}{\underset{︸}{\left(p_{y0}\cdot {\left(h_{y}(t)\cdot l_{y}\cdot (\frac{y(t{)}^{h_{3}}}{y(t{)}^{h_{3}}+h_{4}})\right)}^{-\alpha_{y}})\cdot h_{y}(t)\cdot l_{y}\cdot (\frac{y(t{)}^{h_{3}}}{y(t{)}^{h_{3}}+h_{4}}\right)}}+\\ \underset{non-market\cdot value\cdot of\cdot endemic\cdot fish}{\underset{︸}{\left(\frac{y(t{)}^{\beta_{0}}}{y(t{)}^{\beta_{0}}+\beta_{1}})\cdot (p_{y0}\cdot {\left(h_{y}(t)\cdot l_{y}\cdot (\frac{y(t{)}^{h_{3}}}{y(t{)}^{h_{3}}+h_{4}})\right)}^{-\alpha_{y}})\cdot h_{y}(t)\cdot l_{y}\cdot (\frac{y(t{)}^{h_{3}}}{y(t{)}^{h_{3}}+h_{4}}\right)}}\\ \underset{\cos t\cdot of\cdot harvesting\cdot endemic\cdot fish}{\underset{︸}{-w_{0}\cdot h_{y}(t{)}^{c_{c0}}}}\underset{\cos t\cdot of\cdot harvesting\cdot catfish}{\underset{︸}{-w_{0}\cdot h_{x}(t{)}^{c_{c1}}}} \end{array}\right)}=\mu_{x}(l_{x}\cdot (\frac{x(t{)}^{h_{1}}}{x(t{)}^{h_{1}}+h_{2}})).$$ (A2)

The optimality condition in Eq (A2) requires that the loss in utility to the lake manager from costs incurred through a marginal control effort must equal the effect of that effort on the shadow price of the stock of the invasive fish species. As the effectiveness of the control effort is population dependent, a low stock of exotic fish species may discourage control efforts through increasing their costs.

The first order condition with respect to the harvesting of the endemic fish for economic gains is given as:

$$\frac{-c_{c1}\cdot w_{0}\cdot h_{y}(t{)}^{c_{c1}-1}+\frac{\partial }{\partial h_{y}(t)}(\begin{array}{c} \underset{revenue\cdot from\cdot endemic\cdot fish}{\underset{︸}{\left(p_{y0}\cdot {\left(h_{y}(t)\cdot l_{y}\cdot (\frac{y(t{)}^{h_{3}}}{y(t{)}^{h_{3}}+h_{4}})\right)}^{-\alpha_{y}})\cdot h_{y}(t)\cdot l_{y}\cdot (\frac{y(t{)}^{h_{3}}}{y(t{)}^{h_{3}}+h_{4}}\right)}}+\\ \underset{non-market\cdot value\cdot of\cdot endemic\cdot fish}{\underset{︸}{\left(\frac{y(t{)}^{\beta_{0}}}{y(t{)}^{\beta_{0}}+\beta_{1}})\cdot (p_{y0}\cdot {\left(h_{y}(t)\cdot l_{y}\cdot (\frac{y(t{)}^{h_{3}}}{y(t{)}^{h_{3}}+h_{4}})\right)}^{-\alpha_{y}})\cdot h_{y}(t)\cdot l_{y}\cdot (\frac{y(t{)}^{h_{3}}}{y(t{)}^{h_{3}}+h_{4}}\right)}} \end{array})}{\left(\begin{array}{c} \underset{revenue\cdot from\cdot endemic\cdot fish}{\underset{︸}{\left(p_{y0}\cdot {\left(h_{y}(t)\cdot l_{y}\cdot (\frac{y(t{)}^{h_{3}}}{y(t{)}^{h_{3}}+h_{4}})\right)}^{-\alpha_{y}})\cdot h_{y}(t)\cdot l_{y}\cdot (\frac{y(t{)}^{h_{3}}}{y(t{)}^{h_{3}}+h_{4}}\right)}}+\\ \underset{non-market\cdot value\cdot of\cdot endemic\cdot fish}{\underset{︸}{\left(\frac{y(t{)}^{\beta_{0}}}{y(t{)}^{\beta_{0}}+\beta_{1}})\cdot (p_{y0}\cdot {\left(h_{y}(t)\cdot l_{y}\cdot (\frac{y(t{)}^{h_{3}}}{y(t{)}^{h_{3}}+h_{4}})\right)}^{-\alpha_{y}})\cdot h_{y}(t)\cdot l_{y}\cdot (\frac{y(t{)}^{h_{3}}}{y(t{)}^{h_{3}}+h_{4}}\right)}}\\ \underset{\cos t\cdot of\cdot harvesting\cdot endemic\cdot fish}{\underset{︸}{-w_{0}\cdot h_{y}(t{)}^{c_{c0}}}}\underset{\cos t\cdot of\cdot harvesting\cdot catfish}{\underset{︸}{-w_{0}\cdot h_{x}(t{)}^{c_{c1}}}} \end{array}\right)}=\mu_{y}\cdot l_{y}\cdot \left(\frac{y(t{)}^{h_{3}}}{y(t{)}^{h_{3}}+h_{4}}\right).$$ (A3)

The optimality condition with respect to endemic fish species harvesting requires that the net marginal benefits obtained through fishing revenues must equal the stock adjusted shadow price of the endemic fish species. The arbitrage conditions further determine the shadow price dynamics, however, given the non-linear functional forms, it is not possible derive analytical results.

The current value Hamiltonian for the optimization problem in Eq (12) can be written as:

$$\begin{array}{l} \log\left(\begin{array}{l} p_{y}(t)\cdot h_{y}(t)\cdot l_{y}\cdot (\frac{y(t{)}^{h_{3}}}{y(t{)}^{h_{3}}+h_{4}})+(\frac{y(t{)}^{\beta_{0}}}{y(t{)}^{\beta_{0}}+\beta_{1}})\cdot p_{y}(t)\cdot h_{y}(t)\cdot l_{y}\cdot (\frac{y(t{)}^{h_{3}}}{y(t{)}^{h_{3}}+h_{4}})\\ -w_{0}\cdot h_{y}(t{)}^{c_{c0}}-w_{0}\cdot h_{x}(t{)}^{c_{c1}} \end{array}\right)\cdot \exp(-\lambda (t))+\\ V_{a}(x,y)\cdot \exp(-\lambda (t))\cdot \lambda_{0a}\cdot \frac{x(t{)}^{\phi_{0}}}{x(t{)}^{\phi_{0}}+\phi_{1}}+\mu_{x}\left(\rho_{x}\cdot x(t)\cdot (1-\frac{x(t)}{k_{x}})-(h_{x}(t)\cdot l_{x}\cdot (\frac{x(t{)}^{h_{1}}}{x(t{)}^{h_{1}}+h_{2}}))\right)+\\ \mu_{y}(\rho_{y}\cdot y(t)\cdot (1-\frac{y(t)+\beta \cdot x(t)}{k_{y}})-(h_{y}(t)\cdot l_{y}\cdot (\frac{y(t{)}^{h_{3}}}{y(t{)}^{h_{3}}+h_{4}})))+\mu_{\lambda }\cdot \left(\lambda_{0a}\cdot \frac{x{\left(t\right)}^{\phi_{0}}}{x{\left(t\right)}^{\phi_{0}}+\phi_{1}}\right), \end{array}$$ (A4)

where *μ*_*λ*_ is the shadow price of the stock of the hazard of community adaptation. The first order condition with respect to endemic species harvesting effort is given as:

$$\frac{-c_{c0}\cdot w_{0}\cdot h_{x}(t{)}^{c_{c0}-1}+(1+(\frac{y(t{)}^{\beta_{0}}}{y(t{)}^{\beta_{0}}+\beta_{1}}))\cdot (p_{y}(t)\cdot l_{y}\cdot (\frac{y(t{)}^{h_{3}}}{y(t{)}^{h_{3}}+h_{4}}))}{\left(p_{y}(t)\cdot h_{y}(t)\cdot l_{y}\cdot (\frac{y(t{)}^{h_{3}}}{y(t{)}^{h_{3}}+h_{4}})+(\frac{y(t{)}^{\beta_{0}}}{y(t{)}^{\beta_{0}}+\beta_{1}})\cdot p_{y}(t)\cdot h_{y}(t)\cdot l_{y}\cdot (\frac{y(t{)}^{h_{3}}}{y(t{)}^{h_{3}}+h_{4}})-w_{0}\cdot h_{y}(t{)}^{c_{c0}}-w_{0}\cdot h_{x}(t{)}^{c_{c1}}\right)}=\mu_{y}\cdot l_{y}\cdot \left(\frac{y(t{)}^{h_{3}}}{y(t{)}^{h_{3}}+h_{4}}\right)\cdot \exp(-\lambda (t)).$$ (A5)

The first order condition with respect to endemic fish species harvesting effort requires that the net marginal benefits of harvesting must equal the hazard and stock adjusted shadow price of the endemic fish species population abundance. The shadow price of reducing the stock of the endemic fish species marginally would be higher at lower endemic stock levels, as this would make the crowding out effect from the exotic species more damaging to its future growth. Additionally, a high probability of adaptation (the last term on the right-hand side of Eq A5) would lower the shadow price of reducing endemic population marginally as the post-adaptation scenario is imminent.

First order condition with respect to exotic fish species control effort is similarly given as:

$$\frac{-c_{c0}\cdot w_{0}\cdot h_{y}(t{)}^{c_{c0}-1}}{\left(p_{y}(t)\cdot h_{y}(t)\cdot l_{y}\cdot (\frac{y(t{)}^{h_{3}}}{y(t{)}^{h_{3}}+h_{4}})+(\frac{y(t{)}^{\beta_{0}}}{y(t{)}^{\beta_{0}}+\beta_{1}})\cdot p_{y}(t)\cdot h_{y}(t)\cdot l_{y}\cdot (\frac{y(t{)}^{h_{3}}}{y(t{)}^{h_{3}}+h_{4}})-w_{0}\cdot h_{y}(t{)}^{c_{c0}}-w_{0}\cdot h_{x}(t{)}^{c_{c1}}\right)}=\mu_{x}\cdot \left(l_{x}\cdot (\frac{x(t{)}^{h_{1}}}{x(t{)}^{h_{1}}+h_{2}})\right)\cdot \exp(-\lambda (t)).$$ (A6)

The above condition requires that the marginal costs of exotic fish species control (given by the term on the left-hand side of Eq A6) must equal the weighted shadow price of the stock of the fish species, where the weighting term is the probability of adaptation occurring after time *t* and the stock abundance of the exotic fish species. When the stock of the exotic fish species is low, *exp*(−*λ*(*t*)) would be high, which would raise the cost of letting the stock of the exotic fish species increase marginally. Whereas, when *x*(*t*) is high, *exp*(−*λ*(*t*)) would be low and the cost of an incremental increase in exotic stock would not change much as adaptation is imminent.

## Supporting information

S1 Appendix Table A: Parameter values selected for the numerical example.
(DOCX)
