# Supplementary material for: Protecting endemic species from African Catfish invasion when community behavioral responses get in the way
Source: PLoS One. 2018 Dec 27;13(12):e0209009. doi: 10.1371/journal.pone.0209009 (PMC6307778; doi:10.1371/journal.pone.0209009)
Supplement: S1 Appendix — Table A: Parameter values selected for the numerical example. (DOCX) [file pone.0209009.s001.docx]

**S1 Appendix: Table A: Parameter values selected for the numerical example**

| Equation | Parameter Values | Equation No. |
| --- | --- | --- |
|  | $\rho_{x}$= 0.5; $k_{x}$ = 65,000 | (1) |
| $q_{x}(t)=h_{x}(t)\cdot l_{x}\cdot\left(\frac{x(t)^{h_{1}}}{x(t)^{h_{1}}+h_{2}}\right)$ | $h_{1}$ = 2;$h_{2}$ = 1,000; $l_{x}$ = 0.6 | (2) |
|  | $\rho_{y}$ = 0.1; $k_{y}$ = 65,000; $\beta$ = 2 | (3) |
| $q_{y}(t)=h_{y}(t)\cdot l_{y}\cdot\left(\frac{y(t)^{h_{3}}}{y(t)^{h_{3}}+h_{4}}\right)$ | $h_{3}$ = 2; $h_{4}$ = 1,000; $l_{y}$ = 0.3 | (4) |
| $f_{y}(t)=w_{0}\cdot h_{y}(t)^{c_{c0}}$ | $w_{0}$ = 0.3; $c_{c0}$ = 1.2 | (5) |
| $f_{x}(t)=w_{0}\cdot h_{x}(t)^{c_{c1}}$ | $w_{0}$ = 0.3; $c_{c1}$ = 1.2 | (6) |
| $p_{y}=p_{y0}\cdot\left(h_{y}(t)\cdot l_{y}\cdot\left(\frac{y(t)^{h_{3}}}{y(t)^{h_{3}}+h_{4}}\right)\right)^{-\alpha_{y}}$ | $p_{y0}$ = 2.5; $\alpha_{y}$ = 0.03 | (7) |
| $V_{endem}(t)=\left(\frac{y(t)^{\beta_{0}}}{y(t)^{\beta_{0}}+\beta_{1}}\right)\cdot p_{y}(t)\cdot q_{y}(t)$ | $\beta_{0}=2$; $\beta_{1}=10^{6}$ | (8) |
|  | $\lambda_{0a}$ = 0.1; $\phi_{0}$ = 2; $\phi_{1}$ = 10^6^ | (10) |
| $V_{a}(x,y)$, base case risk | $V_{a}(x,y)=86.263\cdot y(t)^{0.0357}\cdot\exp(-0.0001734\cdot x(t))$ | (11) |
| $V_{a}(x,y)$, loss of endemic value | $V_{a}(x,y)=36.301\cdot y(t)^{0.0383}\cdot\exp(-0.0000273\cdot x(t))$ | (12) |
